# Chronic schistosomiasis in African immigrants in Israel: Lessons for the non-endemic setting

**DOI:** 10.1097/MD.0000000000018481

**Published:** 2019-12-27

**Authors:** Yael Paran, Ronen Ben-Ami, Boris Orlev, Ora Halutz, Ofir Elalouf, Asaf Wasserman, Ofer Zimmerman, Ido Nahmani, Liane Rabinowich, Talya Finn

**Affiliations:** a Infectious Diseases Unit; b Microbiology Laboratory; c Department of Rheumatology; d Department of Surgery; e Department of Gastroenterology; f Sackler Faculty of Medicine, Tel Aviv University, Tel Aviv; g Sanz Medical Centre, Laniado Hospital, Netanya; h Ruth and Bruce Rappaport Faculty of Medicine, Technion Israel Institute of Technology, Haifa, Israel.

**Keywords:** colitis, eosinophilia, immigrants, schistosomiasis, splenomegaly

## Abstract

To study the clinical presentation of Chronic Schistosomiasis (CS) in immigrants from East Africa to Israel and the tests that were useful in confirming the diagnosis.

A retrospective study of all medical notes pertaining to hospitalized patients who were immigrants from East Africa with a pathological or microscopic confirmation of CS. Literature review was also conducted focusing on diagnosis of schistosomiasis among immigrants from endemic countries.

We identified 32 suspected and 11 confirmed cases of CS. Most of the patients (82%) presented with gastrointestinal symptoms. Sensitivity of stool smear, serology and tissue diagnosis (by histopathology or microscopy) were 14%, 100%, 89%, respectively. Patients have undergone extensive diagnostic evaluation with long hospitalization stays (median 10 days, range 4 to 33 days).

CS has multiple presentations and is seen in Israel among refugees from Eritrea and Sudan. Most of the manifestations are gastrointestinal, suggestive of infection with *Schistosoma mansoni (S. mansoni)*. Standard diagnostic techniques used in endemic countries, such as microscopy for ova and parasites were unhelpful, necessitating more advanced procedures like colonoscopic or liver biopsy. We propose a diagnostic algorithm for CS in this patient population in order to make an accurate diagnosis and avoid unnecessary invasive procedures.

## 1 Introduction

Chronic schistosomiasis (CS) is highly prevalent in East Africa, but uncommon in most industrialized countries. In the last decade, immigration from East Africa into Israel has risen sharply, with tens of thousands of refugees arriving from Sudan and Eritrea. By the end of December 2017 there were at least 26,563 Eritreans (71%) and 7624 (21%) Sudanese refugees living in Israel.^[1]^ As with the current immigration into Europe, refugees have been identified as a risk group for chronic infectious diseases including enteric parasitosis, tuberculosis, vivax malaria and CS.^[2]^ However, systematic assessment of the prevalence of these diseases among immigrants is lacking.^[3]^

The Tel Aviv Sourasky Medical Center (TASMC) is a tertiary level hospital serving the metropolitan Tel Aviv area, where most of the Eritrean and Sudanese immigrants reside. Here, we summarize our experience treating immigrants with CS. We highlight characteristic clinical features and difficulties in diagnosing CS in non-endemic countries. Finally, we propose a diagnostic algorithm, which is currently implemented in our hospital.

Investigating the disease in this population may help health practitioners to provide a better care to this unique patient population. This is especially important nowadays since immigration from African countries to European countries is increasing.

## 2 Materials and methods

We retrospectively searched the TASMC Laboratory database for hospitalized patients with a positive diagnostic test result for *Schistosoma spp.* from January 2011 through December 2015.

Demographic and clinical data were extracted from electronic medical records (country of origin, previous diagnosis and treatment, time of immigration, duration of symptoms).

We used the following case definition of CS in immigrants from Africa:

1. A compatible clinical syndrome: hepatic or hepatosplenic enlargement with or without portal hypertension, colitis, or urinary tract schistosomiasis.
2. Microbiological evidence of *Schistosoma spp*. infection, by one of the following:
  a. Detection of *Schistosoma* ova on microscopic examination of the stool or urine;
  b. Observation of *Schistosoma* ova on a pathology specimen of a clinically involved site.
  c. Detection of *Schistosoma* DNA in involved tissue by polymerase chain reaction (PCR) (the PCR used is a RT-PCR which targets the internal transcription spacer 2 (ITS2) gene).^[4]^

Serology was done using a non-species specific *Schistosoma* IgG (Schistosoma Antibody Detection Test, Scimedx Corporation).

We performed a literature review to ascertain the current knowledge regarding various diagnostic modalities for CS. Specifically, we searched for articles that assessed the diagnostic value of serology, stool smears, and colonoscopy.

The study was approved by the Tel Aviv Medical Center Institutional Review Board.

## 3 Results

We identified 32 patients with a positive laboratory test for *Schistosoma spp*: 28 with positive Schistosoma IgG, one with ova detected by stool microscopy, 10 with ova detected on a pathological specimen, and 1 with positive PCR from stool. Of these, 11 patients met the case definition for CS (Table 1).

**Table 1:**
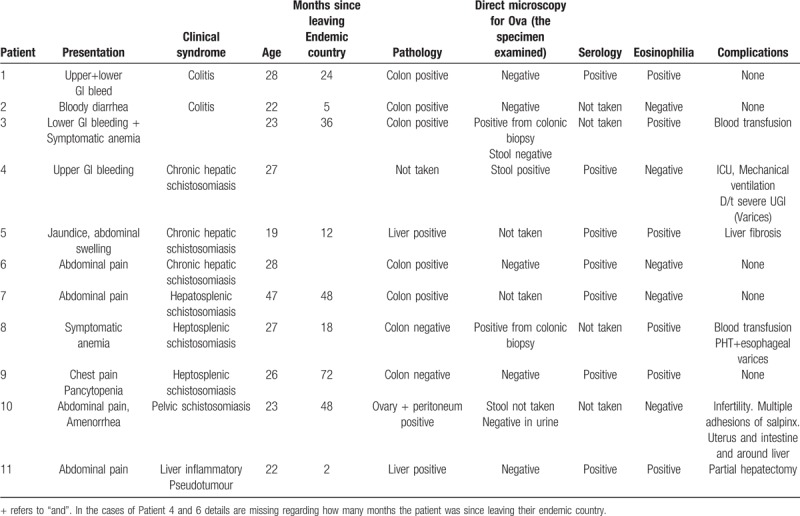
Clinical and epidemiological characteristics, diagnostic tests, and complication of Patients with symptomatic CS.

Patients who did not have a clinical syndrome compatible with CS were considered asymptomatic (patients with incidental finding of peripheral eosinophilia who were hospitalized for an unrelated reason, e.g., orbital trauma).

Patients with CS had a median age of 26 (range 19 to 47 years). Ten (91%) were males. Seven were from Sudan and 4 were from Eritrea. Median time elapsed from immigration to diagnosis was 24 months (range 2–72 months).

Most of the patients (82%) presented with gastrointestinal symptoms such as GI bleeding or abdominal pain. Two (18%) presented with symptomatic anaemia and one patient (9%) presented with pleuritic chest pain due to pneumonia with an incidental finding of pancytopenia.

Eosinophilia was present in 5 of 11 patients (45%). Serology was positive in 7 out of 7 patients tested (100%). Stool microscopy for ova and parasites was positive only in one out of 7 tested patients (14%). Tissue biopsy was performed in 10 patients and 9 out of the 10 biopsies (89%) were positive for schistosomiasis ova (either by pathology (8/9) or by direct microscopy for ova (1/9)): 7 had a colonic biopsy via colonoscopy (6 positive, 86%), 2 had a liver biopsy (2 positive, 100%), 1 had a positive ovarian biopsy (100%) and 1 had a positive peritoneal biopsy (100%).

Hepatic or heptosplenic schistosomiasis were the most frequent clinical syndromes, found in 6 of 11 (55%) patients. Three patients (27%) had colitis, one had pelvic schistosomiasis (9%) and one patient had an inflammatory pseudotumour of the liver (9%).

Five patients experienced a complicated course during hospitalization: two patients required blood transfusions, 2 underwent surgery (explorative laparotomy, partial hepatectomy) and one had severe upper gastrointestinal bleeding from varices requiring admission to the intensive care unit and mechanical ventilation.

**Figure 1:**
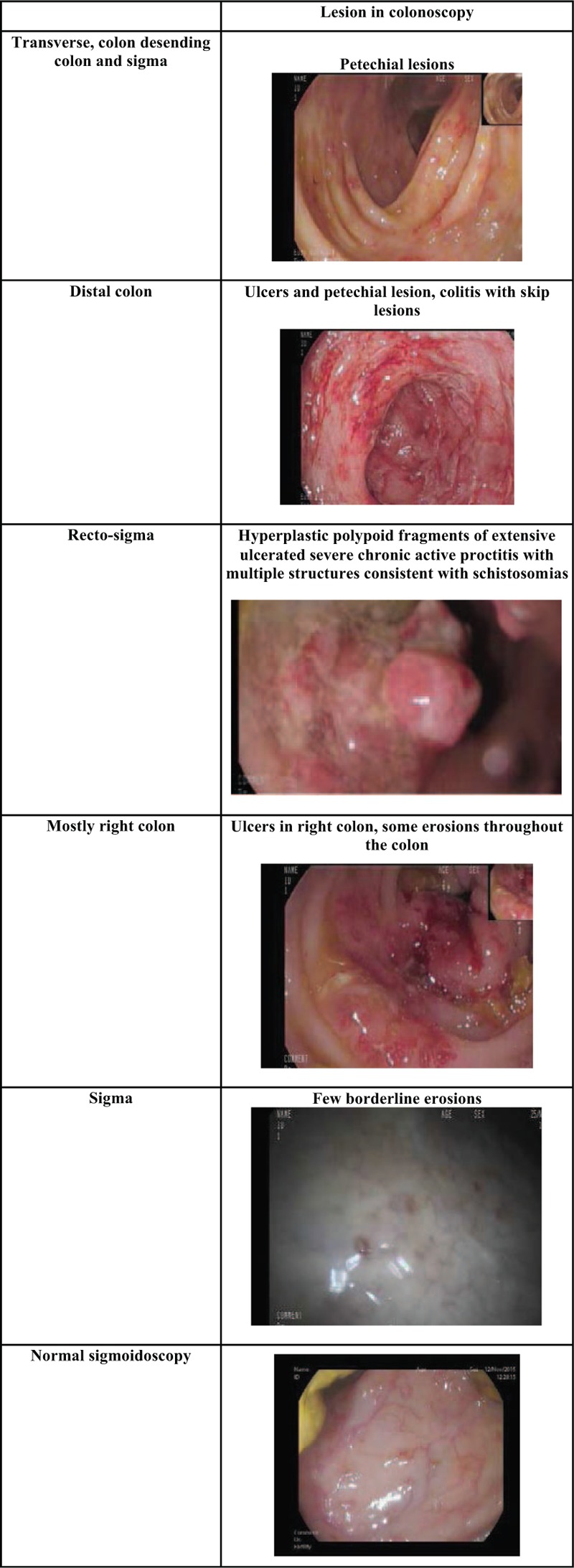
Findings on colonoscopy including images of the types of lesions seen and details of where the biopsies were taken from. Each picture has a brief description above it describing the lesions seen in the images.

Seven patients underwent colonoscopy (Fig. 1). Macroscopic findings were observed in 5 patients (71%): ulcers and erosions in 2 patients, petechial lesions in 2, and large bleeding polypoid masses in one. In all 5 patients lesions were present in the distal colon or the recto-sigmoid area, but some also had lesions in the right or transverse colon. Two patients had a normal macroscopic examination; in 1 patient ova were detected on mucosal biopsy and in 1 stool sample (PCR) was positive for *Schistosoma spp*. DNA.

All patients underwent extensive diagnostic work up with prolonged hospital stay (Table 2). The length of hospitalization ranged from 5 to 33 days with a median of 10 days. The work up included various blood tests including various serologic test for infectious etiologies and autoimmune diseases, microbiological tests including cultures for bacteria, tuberculosis, leishmaniasis and other infectious diseases (Table 2). Most of the patients underwent abdominal CT, chest CT or both colonoscopy and gastroscopy as part of their diagnostic work up. Several patients underwent different invasive procedures such as paracentesis, bronchoscopy, or laparoscopic hepatectomy.

**Table 2:**
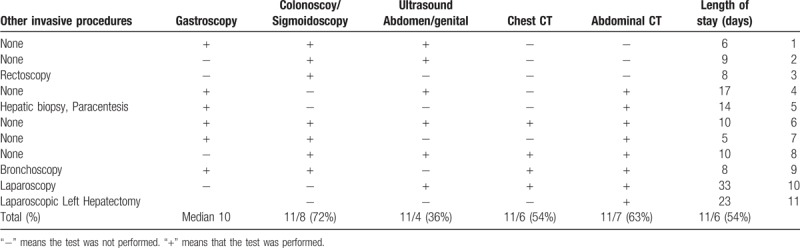
Hospitalization variable. Length of stay (LOS) and diagnostic work up.

All patients were treated with Praziquantel during their hospitalization, and were discharged in good clinical condition. Although they were offered clinical monitoring at an infectious diseases clinic, none of these patients returned for follow-up.

## 4 Discussion

In light of the global trend of increasing migration of individuals from Africa in recent decades knowledge of diseases endemic in these places is becoming increasingly important. CS is a neglected tropical disease. It is estimated that there are 190 million cases of schistosomiasis in sub-Saharan Africa.^[5]^

Due to the immigration of refugees from East Africa, especially from Eritrea and Sudan to Israel over the last few years we have seen several cases of CS.

Unlike acute schistosomiasis, which occurs among young people from endemic areas and travelers, CS is the most common presentation seen among adults living in endemic areas. About 85% of the global cases of schistosomiasis occur in Africa.^[5]^ There is sparse data on the prevalence of CS in Sudan and Eritrea. In Eritrea, *S mansoni* is endemic in certain areas[^6^,^7^]. *Schistosoma haematobium* (*S haematobium*) and *S mansoni* are endemic throughout Sudan except for the Red Sea province.

CS is primarily due to a granulomatous response to the ova deposited in various organs. The granulomatous response is later replaced by fibrosis, which results in many of the symptoms. CS is often a multi-organ disease due to the distribution of eggs and ensuing fibrosis. Infection with schistosomiasis is often asymptomatic until late in the disease. CS can manifest in many ways and have a significant impact on morbidity. The most common syndromes are urinary schistosomiasis, intestinal schistosomiasis and hepatic schistosomiasis. Less common syndromes are neuroschistosomiasis and genital schistosomiasis.^[8]^

This study investigated symptomatic cases with pathological or microbiological evidence of CS among immigrants from East Africa. Patient may present with various manifestation from abdominal pain, gastro intestinal bleeding and symptomatic anemia. We found various clinical syndromes including intestinal CS, hepatosplenic schistosomiasis, pelvic schistosomiasis and liver inflammatory pseudotumor.

This is in contrast to the findings of Clerinx et al, who found that urinary signs and symptoms are the most common manifestation of schistosomiasis in immigrants.^[8]^ This difference can probably be accounted for depending on the various *Schistosoma spp* prevalent in each particular country. In our cohort all the patients with diagnosis to the specie level (4 patients) were found to have *S mansoni.* Since most of the diagnoses were based on eggs seen in histopathology tissue examination and not direct microscopy the diagnosis of the exact *Schistosoma spp*. was not definite in most of the patients. However, in light of the fact that cases in Eritrea and Sudan are known to be caused by *S mansoni* (that causes GI schistosomiasis) and *S haematobium* (that causes genitourinary schistosomiasis)[^6^,^7^] and all but one case were gastrointestinal we believe that all the cases in our cohort were most probably due to *S mansoni*. Even the case that was not gastrointestinal was confirmed as *S mansoni*.

Inflammatory pseudo-tumour of the liver appears to be a rare manifestation. To our knowledge this is the first reported case in the liver in an adult patient.

The young age range of the patients in our study (19–47 years) and the male predominance is representative of the age range of the immigrant community in Israel.

None of the patients participated in follow up underscoring the challenges of continuity of care in patients who do not have medical insurance.

Despite standard methods of practice whereby stool microscopy is the diagnostic method of choice, in our institution serology appeared to be a more sensitive method for the diagnosis of schistosomiasis. There was stool evidence of *S mansoni* in 14% (1/7 cases) while serology had 100% sensitivity (7/7). This is compatible with previous studies showing low sensitivity for stool microscopy in immigrants from endemic countries. Miller et al found a high sero-prevelence rate (73%) amongst immigrants from Somalia to the USA with only 2% out of 390 Somali immigrants screened found to have ova of *S hematobium* in urine, suggesting low worm burden.^[9]^ Posey et al also found high schistosomiasis seroprevalence during screening of “lost boys and girls” of Sudan and Somali Banto who immigrated to the USA, 44% and 73% respectively.^[10]^ Therefore for immigrants from endemic countries, especially those arriving in industrialized countries less familiar with CS, serology could serve as a useful screening tool for CS. However, serology does not allow for differentiation between acute or chronic disease. It may remain positive after treatment and for this population from endemic countries it does not imply causality.^[10]^

Whithy et al found a sensitivity for serology of 86%, 951 positive out of 1107 patients and an sensitivity for ova detection (stool and urine) of 45% but the cohort included travelers and not only immigrants.^[11]^

We hypothesize that the low sensitivity of stool microscopy in this population could be due to a low parasite load in those who left the endemic country months or years before the stool was tested because some adult worms may have died reducing ova secretion. Another explanation could be that laboratory personal in non-endemic countries are less experienced in making a microscopic diagnosis.

**Figure 2:**
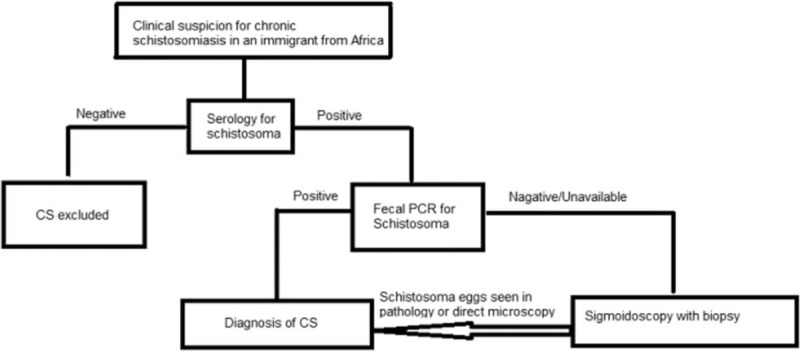
A diagnostic algorithm for a patient suspected of having Chronic Schistosomiasis (CS) explaining the different stages involved in diagnosis whether it be serology, PCR or pathological diagnosis of CS.

Colonoscopy and liver biopsy were shown to be useful tools for diagnosing the disease. Colonoscopy with colon biopsy proved diagnostic even in cases where the patient did not have any symptoms of colitis. Our experience suggests the potential importance of performing a colonoscopic biopsy in patients with unexplained hepato-splenomegaly and relevant epidemiology (Fig. 2). This is also compatible with previous studies.

Biopsy from the colon, rectum or other organs involved in the disease appears to be important in the diagnosis of CS when other tests are negative. This observation was also shown by Harries et al who used the rectal snip test to diagnose CS in expatriates. Rectal biopsy led to the diagnosis of 50% out of 173 patients evaluated.^[12]^ Yasawy et al compared biopsies from sigmoidoscopy and colonoscopy to fecal smears and serology. Only 11.4% with biopsy proven CS had positive stool smear.^[13]^

Whilst this was not used in most of our cases, PCR from fecal samples appears to have an excellent sensitivity and specificity for diagnosis of CS. Abdel –Hafeez found the fecal PCR for schistosoma chronic intestinal schistosomiasis was superior to ova detection in stool. This superiority was much more pronounced for chronic infection than for acute since the egg deposition in chronic cases is much lower. In their study in the chronic intestinal schistosomiasis group, the percentage of positive cases detected by the Kato-Katz method was 8.6%, while that of PCR was 97.1%.^[14]^

From the cases that we reviewed, we conclude that there do not appear to be specific macroscopic characteristics on colonoscopy that can be deemed “typical” of CS (Fig. 1). Both macroscopic appearance and lesion location were diverse on colonoscopy. The type of lesion seen ranged from large bleeding polypoid masses to ulcers to minimal erosions or a completely normal colonoscopy, even in patients with other tests that indicated colonic involvement (such as the finding of ova in direct microscopy from mucosal biopsy or a positive PCR for schistosoma in stool). The location of the pathological finding within the colon differed between patients. All patients with a pathological examination had involvement of the distal colon or the recto sigmoid region. In another study about colonoscopy findings in CS, Cao et al found the lesion was mainly located in the sigma or rectum in 63%.^[15]^

Interestingly, even when the colonoscopy seemed macroscopically normal or almost normal blind samples were still diagnostic of CS either by pathology or by visualizing ova from colonic mucosa on direct microscopy (Fig. 1, Table 1)

Some of the patients went through an extensive and costly work up with prolonged hospitalizations (median 10 days, range 4 to 33 days) before arriving at the correct diagnosis (Table 2). This is especially true of patients with hepatosplenic schistosomiasis with or without pancytopenia. Their work up included serology for hepatotrophic viruses, antibodies for auto immune liver diseases or abdominal imaging with computed tomography or ultrasound. Some even went through invasive diagnostic procedures such as bone marrow biopsies explorative laparotomy, liver biopsy and even a partial hepatectomy. Some of these procedures might have been unnecessary if a diagnosis of CS could have been established sooner. Our experience has taught us that when treating patients from developing countries physicians in developed countries often apply differential diagnoses relevant to the developed world leaving those diagnoses that may be more relevant such as CS to be considered last.

Based on our experience and the literature we propose the use of an algorithm included as Figure 2, when suspecting CS in an immigrant from East Africa.

CS should be suspected in any patient from endemic country with lower GI bleeding, abdominal pain or in patient with the incidental finding of pancytopenia or an enlarged spleen. Especially in afebrile patients or who lack other constitutional findings which might otherwise suggest alternative diagnoses (such as leishmania or malaria).

In conclusion, Schistosomiasis is an important differential diagnosis when evaluating patients with gastrointestinal manifestations in immigrants from endemic countries. With increasing migration around the world we suspect that CS will become increasingly relevant in people admitted to hospitals in developed countries. Following a simple algorithm presented here can shorten hospital stay, reduce costs and avoid unnecessary and sometime invasive procedures.

## 5 Limitations

This study was a single center study involving immigrants from only 2 countries, Eritrea and Sudan. However, we believe that the findings were significant enough that they can still be generalized for all patients coming from endemic CS. It was a retrospective study with all the limitations of this type of study. It was largely reliant on reviewing medical notes that do not always detail all clinical information. However, all cases had infectious diseases consultation performed for each patient, improving the chance that all significant positive and negative findings were recorded in the notes.

## Author contributions

**Conceptualization:** Yael Paran.

**Data curation:** Yael Paran, Ronen Ben-Ami, Ofer Zimmerman, Liane Rabinowich, Talya Finn.

**Formal analysis:** Yael Paran, Talya Finn.

**Investigation:** Yael Paran, Boris Orlev, Ora Halutz, Asaf Wasserman, Talya Finn, Ofir Elalouf, Ido Nahmani.

**Methodology:** Yael Paran, Talya Finn.

**Project administration:** Yael Paran.

**Writing – original draft:** Yael Paran, Talya Finn.

**Writing – review & editing:** Yael Paran, Ronen Ben-Ami, Ora Halutz, Ofir Elalouf, Asaf Wasserman, Ofer Zimmerman, Ido Nahmani, Talya Finn.

Talya Finn orcid: 0000-0001-7955-8725.
